# RETRACTION: Bu Shen Yi Sui Capsules Promote Remyelination by Regulating MicroRNA‐219 and MicroRNA‐338 in Exosomes to Promote Oligodendrocyte Precursor Cell Differentiation

**DOI:** 10.1155/ecam/9803185

**Published:** 2026-06-15

**Authors:** Evidence-Based Complementary and Alternative Medicine

RETRACTION: J. Ji, Y. Q. Sun, Z. Zha, et al. “Bu Shen Yi Sui Capsules Promote Remyelination by Regulating MicroRNA‐219 and MicroRNA‐338 in Exosomes to Promote Oligodendrocyte Precursor Cell Differentiation,” *Evidence-Based Complementary and Alternative Medicine 2022*, no. 1 (2022): 3341481, https://doi.org/10.1155/2022/3341481.

The above article, published online on 13 April 2022 in Wiley Online Library (https://onlinelibrary.wiley.com/), has been retracted by agreement between the authors and John Wiley & Sons Ltd. The retraction has been agreed after the authors requested a correction to address the following errors:

- In Figure 1 (c), the panels of NF160 in spinal cord for EAE + PA and EAE + BSYSC are duplicated;
- In Figure 7 (d), the panel for Ad. miR‐219‐5p‐sponge + exo‐BSYSC is incorrect.

During the journal’s investigation, the following additional concerns were noted:

- The B‐actin blots in Figure 6 share unexpected similarities;
- There are unexpected similarities between panels in Figure 7(a).

The authors disagree that the similarities noted in Figures 6 and 7(a) are of concern. The authors stated that several target proteins and internal references were detected on the same membrane, which resulted in the described Figure 6 similarities.

The authors also stated that the partial overlap between the NC + Exo‐BSYSC group in the miR‐219 and miR‐338 panels in Figure 7 (a), are due to these panels belonging to the same control group, under the same experimental conditions, and are only displayed separately.

The authors were not able to provide all of their raw data, as some data were inaccessible due to damage of storage devices. As such, the authors have requested a retraction, as the results can no longer be verified. The publisher agrees with the retraction.

